# Supplementary material for: Exponential quantum advantages in learning quantum observables from classical data
Source: npj Quantum Inf. 2026 Jan 10;12(1):19. doi: 10.1038/s41534-025-01162-2 (PMC12846916; doi:10.1038/s41534-025-01162-2)
Supplement: Supplementary file 1 — Supplementary Information [file 41534_2025_1162_MOESM1_ESM.pdf]

Supplementary information for "Exponential quantum advantages in learning quantum observables from classical data"

1 Related work

Although we are not the first to prove learning advantages for BQP-complete problems, our results present significant differences compared to previous works.

In [1] the authors first suggested that many learning speed-ups could arise by looking at BQP-complete problems. However, there are at least two crucial differences with our work. Firstly, the classical hardness result in [1] is derived by addressing heuristic complexity classes found in distributional problems. Specifically, it is assumed that there exists an input distribution such that a BQP-complete language $\mathcal{L}$ cannot be heuristically solved by a classical algorithm, i.e., $(\mathcal{L}, \mathcal{D}) \not\subseteq$ HeurP/poly. In contrast, in this work we employ a different proof technique and base all of our separation results on the assumption that BQP $\not\subseteq$ P/poly, thus focusing on the more widely investigated complexity classes of decision problems. Secondly, the learning problems considered in [1] have a concept class of polynomial size, and the quantum algorithm crucially leverages this to find the underlying concept in polynomial time by brute force. In this work we take a step further from their result by considering a physically relevant instantiation of a BQP-complete function. The associated concept classes are continuous and the quantum learning algorithm learns the unknown concept from data through a natural machine learning method.

In recent works by [2, 3], significant progresses have been made with classical machine learning algorithms with provable guarantees for learning ground state properties from data, restricting the role of a quantum computer only to the data acquisition phase. In this study, we establish rigorous limitations on the capabilities of classical algorithms when learning quantum observables. A key distinction from their approach is that, in considering ground state problems, we investigate Hamiltonians with a polynomially decaying spectral gap, whereas the algorithms in [2, 3] have provable guarantees only for gapped Hamiltonians. The works in [4, 5] significantly extended the set of classical learnable quantum states to states which lies in the same Lindbladian phase. With respect to their works, our findings establish a definitive boundary on what can be learned through classical methods.

In [6] the authors proposed a family of supervised learning tasks which present a learning speed-up with an exponentially sized concept class. Our construction, which was developed independently from [6], stems from a physically-motivated context, resulting in the utilization of different techniques for the quantum learning algorithm given the necessity to handle errors in the training samples. Furthermore, their complexity results are, once again, derived by focusing on distributional problems rather than decision problems.

Finally, in [7], the authors provide bounds on the overhead in training samples required by a classical algorithm compared to a quantum algorithm for the task of predicting a fixed observable. In contrast, our results focus primarily on the time efficiency of the learning algorithm. Furthermore, the results in [7] assume a fixed target distribution, whereas our setting requires learnability across all input distributions.

2 Discussion on the assumption $\mathsf{BQP} \not\subseteq \mathsf{P/poly}$

The exponential advantage proposed in this paper is based on the assumption that $\mathsf{BQP} \not\subseteq \mathsf{P/poly}$. The assumption is widely accepted in the complexity community. For example, since factoring and discrete logarithms lie in BQP (via Shor's algorithm), RSA and Diffie–Hellman are breakable by a polynomial time quantum algorithm. If $\mathsf{BQP} \subseteq \mathsf{P/poly}$ then every BQP algorithm admits a family of polynomial-size classical circuits, so factoring and discrete logarithm would be in P/poly. Hence there would be nonuniform polynomial-time (circuit) attacks on RSA and Diffie–Hellman. In oder words, well-established and widely used cryptosystems like Diffie-Helmann and RSA would be insecure under standard definitions. That contradicts contemporary cryptographic standards [8], where baseline security is defined against nonuniform PPT adversaries, captured by P/poly. Finally, we also note that for sampling problems further evidence are available. In particular, as sketched by Aaronson in [9], if $\mathsf{SampBQP} \subseteq \mathsf{SampBPP/poly}$, then this could cause the polynomial hierarchy to collapse.

3 Proof of classical hardness for Theorem 1 in the main text

As anticipated in the main text, in order to prove our main Theorem 1 we need to show both classical hardness and quantum learnability for the time-evolution learning problem. In this section, we prove Theorem 4 for the time-evolution learning problem, formally described through the concept class $\mathcal{F}^{H,O}_{\text{evolved}}$. It is important to notice that, as we discussed in the main text, the same proofs can be easily extended for the class $\mathcal{F}^{\mathcal{H},O}_{\text{g.s.}}$ of measurements on ground state of local Hamiltonians through the Kitaev's circuit-to-hamiltonian construction.

3.1 BQP-completeness of constant time-evolution

Before stating the proof for Therem 4, let us prove an useful lemma which guarantees the BQP-completeness of constant time Hamiltonian evolution following the idea in present in [10]. It is important to notice that the Hamiltonians used in Lemma 1 will not have a constant norm. This is however not a problem for our quantum algorithm as their norms will scale only polynomially with the number of qubits.

Lemma 1 (Constant time-evolution is BQP-complete)**.** *For any $k-$gate quantum circuit $U = U_k ... U_2 U_1$ which acts on n qubits there exists a local Hamiltonian H such that for any n qubit inital state $|\psi\rangle$:*

$$e^{iHt} |\psi\rangle |0\rangle = U_k ... U_2 U_1 |\psi\rangle |k\rangle \tag{1}$$

for $t = \pi$.

Proof. Consider the Feyman clock Hamiltonian [11, 12]:

$$H := \sum_{j=1}^{k} H_j \tag{2}$$

where:

$$H_j = U_j \otimes |j\rangle \langle j-1| + U_j^\dagger \otimes |j-1\rangle \langle j| \tag{3}$$

notice that using the common unary encoding for the clock register, such Hamiltonian is 4 local [12]. Furthermore, the Hamiltonian H acts on two different registers: the first register ("work register") consists of n qubit and it will store the computation of the circuit U, the second register ("clock register") contains $k+1$ qubit and it acts as a counter which records the progress of the computation. Now, if we evolve an initial state $|\psi_0\rangle = |\psi\rangle |0\rangle$ under the Hamiltonian H the evolved state will be in the space spanned by the $k+1$ states $\{|\psi_j\rangle = U_j ... U_2 U_1 |\psi\rangle |j\rangle\}_{j=0}^{k}$. After letting the system evolve for a time τ, if now we measure the clock register and obtain a value L then the work register will

exactly contains the computation of the quanutm circuit U on the initial state $|\psi\rangle$. This of course will happen only with a certain probability and while there are many ways to boost such probabilities of getting the desired final state $|\psi_k\rangle = U_k...U_2U_1 |\psi\rangle |k\rangle$, the idea in [10] is to modify the Feynman's Hamiltonian in Eq. (2) in order to make the evolution perfect as in Eq. (1).

Notice first that in the subspace spanned by the vectors $\{|\psi_j\rangle\}_{j=0}^k$ the non zero entries of the matrix H are

$$\langle\psi_j| H |\psi_{j\pm1}\rangle = 1 \tag{4}$$

We now follow the idea in [10] and modify the Hamiltonian in (2) in the following way:

$$H' := \sum_{j=1}^{k} \sqrt{j(k+1-j)}\, H_j \tag{5}$$

The idea in [10] is to associate each state in $\{|\psi_j\rangle\}_{j=0}^k$ to a quantum system of total angular momentum $\frac{k}{2}(\frac{k}{2}-1)$ with z component $j-\frac{k}{2}$. The association is possible by the fact that a system with total angular momentum $\frac{k}{2}(\frac{k}{2}-1)$ will allow $k+1$ states with z components of values $-\frac{k}{2}, -\frac{k}{2}+1, ..., \frac{k}{2}-1, \frac{k}{2}$. These are exactly the $k+1$ states in $\{|\psi_j\rangle\}_{j=0}^k$ and it is possible to move among them defining the corresponding ladder operators :

$$L_- |\psi_j\rangle = \sqrt{j(k+1-j)}\,|\psi_{j-1}\rangle \tag{6}$$

$$L_+ |\psi_j\rangle = \sqrt{(k-j)(j+1)}\,|\psi_{j+1}\rangle \tag{7}$$

By the algebra of the angular momentum, the x component of the total angular momentum J_x can be expressed as

$$J_x = \frac{1}{2}(L_+ + L_-) \tag{8}$$

Comparing Eq.(4) with Eq. (6) it is clear that H' is exactly the x component of the angular momentum operator defined over the states $\{|\psi_j\rangle\}_{j=0}^k$. As J_x rotates between the states with z component $\pm\frac{k}{2}$ in time $t=\pi$, this concludes the proof.

□

3.2 Proof of Theorem 4 in the main text

The second ingredient in demonstrating the classical hardness of our learning task, in addition to the BQP-completeness of time-evolution, is a result from classical learning theory contained in [13]. Here, we will state this result in Lemma 2 and, for clarity and didactical purpose, provide an intuitive explanation of its proof.

Lemma 2 (Learnability implies evaluation - Theorem 7 in [13]). *Suppose $\mathcal{F}$ is learnable in the sense of Def. 1 with input space $\mathcal{X}_n = \{0,1\}^n$. Then there exists a polynomial p such that for all the concepts $f \in \mathcal{F}$ of size s, there exists a classical circuit of size $p(n,s)$ exactly computing f*

Proof sketch. In Lemma 2, the size s of a concept refers to the length of its representation under some encoding scheme. For example, in the case of our concept class $\mathcal{F}_{\text{evolved}}^{H_{\text{hard}},O}$, $s \sim \text{poly}(n)$ as our concepts can be implemented by polynomial size quantum circuits. The key result underlying Lemma 2 is another finding from [13], which states the following. If the concept class $\mathcal{F}$ is learnable in the sense of Def. 1, then there exists a learning algorithm that, for any target concept $f \in \mathcal{F}$, produces a hypothesis implementable by a circuit of size $\text{poly}(n, s, \log(1/\epsilon))$, which is ϵ-close to the target concept in the sense of Eq.1. This remarkable result is achieved through a smart application of boosting theory. The proof of Lemma 2 then follows by applying the boosted learning algorithm to a training set containing all input points $x \in \{0,1\}^n$ and requiring an exponential precision $\epsilon \leq \frac{1}{2^n}$. □

We are now ready to state the proof of Theorem 4 on the existence of a Hamiltonian H_{hard} such that the concept class $\mathcal{F}_{\text{evolved}}^{H_{\text{hard}},O}$ is not classically learnable in the sense of Def. 4.

Theorem (Classical hardness of the time-evolution learning problem)**.** *theorem For any* BQP*-complete language, there exists a Hamiltonian* H_{hard} *such that no randomized polynomial-time classical algorithm* A_c *satisfies the learning condition of Def. 4 for the concept class* $\mathcal{F}^{H_{\text{hard}},O}_{\text{evolved}}$*, unless* $\mathsf{BQP} \subseteq \mathsf{P/poly}$.

Proof. Let $\{U^n_{BQP}\}_n$ be a family of quantum circuits which decides an arbitrary BQP language $\mathcal{L}$, one circuit per size. Precisely, according to the definition of BQP in Def. 2, this implies that for any $\boldsymbol{x} \in \{0,1\}^n$ measuring the first qubit in the computational basis on the state $U^n_{BQP}|\boldsymbol{x}\rangle$ will output 1 (or 0) with probability greater that 2/3 if $\boldsymbol{x} \in \mathcal{L}$ ($\boldsymbol{x} \notin \mathcal{L}$). It is obvious that the the quantum model $f^{O'} = \text{Tr}[\rho(\boldsymbol{x})O']$ correctly decides every $\boldsymbol{x} \in \mathcal{L}$ if $\rho(\boldsymbol{x}) = U^n_{BQP}|\boldsymbol{x}\rangle\langle\boldsymbol{x}|(U^n_{BQP})^\dagger$ and $O' = Z \otimes \underbrace{I \otimes ... \otimes I}_{n-1}$. In fact measuring the first qubit of $U^n_{BQP}|\boldsymbol{x}\rangle$:

1. For all $\boldsymbol{x} \in \mathcal{L}$, $f^{O'}(\boldsymbol{x}) = \Pr$[the output of U^n_{BQP} applied on the input $|\boldsymbol{x}\rangle$ is 1] - Pr[the output of U^n_{BQP} applied to the input $|\boldsymbol{x}\rangle$ is 0] $\geq 2/3 - 1/3 = 1/3$.

2. For all $\boldsymbol{x} \notin \mathcal{L}$, $f^{O'}(\boldsymbol{x}) = \Pr$[the output of U^n_{BQP} applied on the input $|\boldsymbol{x}\rangle$ is 1] - Pr[the output of U^n_{BQP} applied to the input $|\boldsymbol{x}\rangle$ is 0] $\leq 1/3 - 2/3 = -1/3$.

Therefore, as $f^{O'}(\boldsymbol{x}) > 0$ if $\boldsymbol{x} \in \mathcal{L}$ and $f^{O'}(\boldsymbol{x}) < 0$ if $\boldsymbol{x} \notin \mathcal{L}$ such quantum model could efficiently decide the language $\mathcal{L}$. By Lemma 1, for every n there exist a 4-local Hamiltonian H_{hard} such that evolving the state $|\boldsymbol{x}\rangle$ for a time $t = \pi$ under will produce the state $U^n_{\mathsf{BQP}}|\boldsymbol{x}\rangle$ (on the work register). Therefore, the concept class $\mathcal{F}^{H_{\text{hard}},O}_{\text{evolved}}$ contains a concept $f^{\boldsymbol{\alpha'}}$, where $\boldsymbol{\alpha'}$ is defined such that $O(\boldsymbol{\alpha'}) = O'$, which correctly decides $\mathcal{L}$. The final step of the proof lies in noticing that by Lemma 2, if $\mathcal{F}^{H_{\text{hard}},O}_{\text{evolved}}$ is learnable in the sense of Def. 4, then there must exists a polynomial size classical circuit which evaluates $f^{\boldsymbol{\alpha'}}$ correctly on every $\boldsymbol{x} \in \{0,1\}^n$. As $f^{\boldsymbol{\alpha}}$ decides the BQP language $\mathcal{L}$, and for any BQP language we can construct such Hamiltonian H_{hard}, this implies that $\mathsf{BQP} \subseteq \mathsf{P/poly}$.

□

4 Proof of quantum learnability

In this Section we prove that Algorithm 1 satisfies the learning condition in Def. 4 using only polynomial resources in both time and samples. As a first step, notice that the quanutm states $\rho_{H_{\text{hard}}}(\boldsymbol{x})$ in Eq. (3) of $\mathcal{F}^{H_{\text{hard}},O}_{\text{evolved}}$ are easily preparable on a quantum computer. This is due to the fact that the Hamiltonians H_{hard} used to establish our hardness result in Theorem 4 are local, allowing their time-evolution to be efficiently simulated on a quantum computer. Since the quantum algorithm can efficiently prepare time evolved states of local Hamiltonians, the quantum states $\rho_{H_{\text{hard}}}(\boldsymbol{x}) = U|\boldsymbol{x}\rangle\langle\boldsymbol{x}|U^\dagger$ with $U = e^{iH_{\text{hard}}\pi}$ can efficiently be prepared on a quantum computer. In order to demonstrate the quantum learnability of $\mathcal{F}^{H_{\text{hard}},O}_{\text{evolved}}$, all that remains is to rigorously bound the sample and time complexity of Algorithm 1 in the following Theorem 2. As the bound obtained on the sample complexity derives from the generalization bound of the LASSO algorithm [3, 14], we first repeat this result.

Theorem 1 (Theorem 11.16 in [14])**.** *Let* $\mathcal{X} \subseteq \mathbb{R}^A$ *and* $\mathcal{C} = \{\boldsymbol{x} \in \mathcal{X} \mapsto \vec{w} \cdot \boldsymbol{x} : \|\vec{w}\|_1 \leq B\}$*. Let* $\mathcal{S} = ((\boldsymbol{x}_1, y_1), \ldots, (\boldsymbol{x}_N, y_N)) \in (\mathcal{X} \times \mathcal{Y})^N$*. Let* $\mathcal{D}$ *denote a distribution over* $\mathcal{X} \times \mathcal{Y}$ *according to which the training data* $\mathcal{S}$ *is drawn. Assume that there exists* $r_\infty > 0$ *such that for all* $\boldsymbol{x} \in \mathcal{X}$, $\|\boldsymbol{x}\|_\infty \leq r_\infty$ *and* $M > 0$ *such that* $|h(\boldsymbol{x}) - y| \leq M$ *for all* $(\boldsymbol{x}, y) \in \mathcal{X} \times \mathcal{Y}$*. Then, for any* $\delta > 0$*, with probability at least* $1 - \delta$*, each of the following inequalities holds for all* $h \in \mathcal{C}$*:*

$$\begin{aligned} &\mathbb{E}_{(\boldsymbol{x},y)\sim\mathcal{D}}[|h(\boldsymbol{x}) - y|^2] := R(h) \\ &\leq \hat{R}_{\mathcal{S}}(h) + 2r_\infty BM\sqrt{2\log(2|A|)N} + M\sqrt{2\log(1/\delta)/2N} \end{aligned} \tag{9}$$

where $R(h)$ *is the prediction error for the hypothesis* h *and* $\hat{R}_{\mathcal{S}}(h)$ *is the training error of* h *on the training set* $\mathcal{S}$*.*

We are now ready to state Theorem 2 (a rigorous formulation of Lemma 5 in the main text) which provides precise guarantees on the number of samples and the time complexity required by Algorithm 1.

Theorem 2 (Lemma 5 in the main text)**.** *Given n, $\delta > 0$, $\frac{1}{e} > \epsilon > 0$ and a training data set $\mathcal{T}_N^{\boldsymbol{\alpha}} = \{(\boldsymbol{x}_\ell, y_\ell)\}_{\ell=1}^N$ of size*

$$N = \mathcal{O}\left(\frac{\log(\text{poly}(n)/\delta)\text{poly}(n)}{\epsilon^2}\right) \tag{10}$$

where $\boldsymbol{x}_\ell$ is sampled from an unknown distribution $\mathcal{D}$ over $\boldsymbol{x} \in \{0,1\}^n$ and $|y_\ell - Tr(O(\boldsymbol{\alpha})\rho(\boldsymbol{x}_\ell))| \leq \epsilon$ for any geometrically local observable $O(\boldsymbol{\alpha}) = \sum_{i=1}^m \boldsymbol{\alpha}_i P_i$ with $P_i \in \{I, X, Y, Z\}^{\otimes n}$ and $\boldsymbol{\alpha} \in [-1,1]^m$, and $\rho(\boldsymbol{x}) = U(\boldsymbol{x}) |0\rangle \langle 0| U^\dagger(\boldsymbol{x})$. Then there exists a quantum algorithm $\mathcal{A}_q(\mathcal{T}_N^\alpha, \boldsymbol{x}) = h(\boldsymbol{x})$ such that:

$$\mathbb{E}_{x\sim\mathcal{D}}|h(\boldsymbol{x}_l) - Tr[O(\boldsymbol{\alpha})\rho(\boldsymbol{x})]|^2 \leq \epsilon \tag{11}$$

with probability at least $1-\delta$. The computational time of the quantum algorithm is bounded in $\mathcal{O}(\text{poly}(n)N)$

Proof. The proof of the theorem is based on the well known bound on the prediction error of the LASSO algorithm of Theorem 1. Consider the algorithm described in Algorithm 1. First, we demonstrate now that Algorithm 1 satisfies the condition of Theorem 6. In our setting, the input space of the learned model h is $\mathcal{X} = [-1,1]^m \subset \mathbb{R}^m$ as we consider h a function of the $m-$dimensional feature vector $\phi(\boldsymbol{x})$. Clearly, $||\phi(\boldsymbol{x})||_\infty \leq 1$ for all $\boldsymbol{x} \in \mathcal{X}$. The hypothesis class $\mathcal{C}$ of our algorithm is given by the set of functions of the same form of the learned h, i.e. $\mathcal{C} = \{\phi(\boldsymbol{x}) \in \mathcal{X} \to w \cdot \phi(\boldsymbol{x}) : ||w||_1 \leq B\}$ with $B = \text{poly}(n)$. With respect to Theorem 6, we can also choose $M = \text{poly}(n)$ so that $|h(\boldsymbol{x}_l) - y_l| < M$ for all $l = 1, ..., N$:

$$\begin{aligned} |h(\boldsymbol{x}_l) - y_l| &\leq |w \cdot \phi(\boldsymbol{x}_l)| + |y_l| \leq ||w||_1 ||\phi(\boldsymbol{x}_l)||_\infty + 2 \\ &\leq \text{poly}(n) \end{aligned} \tag{12}$$

Where the second inequalitiy follows by Hölder's inequality. By Theorem 1 then, the bound on the prediction error $R(h)$ of the learned model $h(\boldsymbol{x}) = w^*\phi(\boldsymbol{x})$ is

$$R(h) \leq \hat{R}(h) + 2BM\sqrt{\frac{2\log(2m)}{N}} + M^2\sqrt{\frac{\log\delta^{-1}}{2N}} \tag{13}$$

where $\hat{R}(h)$ is the training error on the dataset $T_N^{\boldsymbol{\alpha}}$.

We now bound the training error $\hat{R}(h)$. Let ϵ_1 be the maximum sampling error associated to the measurement of a Pauli observable on $\rho(\boldsymbol{x}_\ell)$, i.e.

$$\epsilon_1 = \max_{\substack{i\in[1,\ldots,m] \\ \boldsymbol{x}\in\{0,1\}^n}} |\text{Tr}[\rho(\boldsymbol{x})P_i] - \phi(\boldsymbol{x}_i)| \tag{14}$$

Let also ϵ_2 the maximum sampling error associated to y_ℓ, such that:

$$|y_\ell - \text{Tr}[O(\boldsymbol{\alpha})\rho(\boldsymbol{x}_l)]| \leq \epsilon_2 \quad \forall (\boldsymbol{x}_\ell, y_\ell) \in T_N^{\boldsymbol{\alpha}} \tag{15}$$

We can now derive a bound on the training error for the optimal value of $w_{opt} = \boldsymbol{\alpha}$ of the model

$h_{opt}(\boldsymbol{x}) = \boldsymbol{\alpha} \cdot \phi(\boldsymbol{x})$:

$$\hat{R}(h_{opt}) = \frac{1}{N}\sum_{\ell=1}^{N} |h_{opt}(\boldsymbol{x}_\ell) - y_\ell|^2 \leq \max_\ell |h(\boldsymbol{x}_\ell) - y_\ell| \tag{16}$$

$$\leq (|\mathrm{Tr}[\rho(\boldsymbol{x}_{\ell^*})O(\boldsymbol{\alpha})] - h(\boldsymbol{x}_{\ell^*})| + |\mathrm{Tr}[\rho(\boldsymbol{x}_{\ell^*})O(\boldsymbol{\alpha})] - y_\ell|)^2 \tag{17}$$

$$\leq \left(\left(\sum_i |\boldsymbol{\alpha}_i|\right)\epsilon_1 + \epsilon_2\right)^2 \tag{18}$$

$$\leq (B\epsilon_1 + \epsilon_2)^2 \tag{19}$$

In practice, we can require to the LASSO algorithm to obtain a w^* which achieves a training error at most $\epsilon_3/2$ larger than the optimal one. We can obtain such precision by setting $B = \mathrm{poly}(n)$. Formally, we have

$$\hat{R}(h) \leq \frac{\epsilon_3}{2} + \min_{\substack{w \in \mathbb{R}^m \\ ||w||_1 \leq B}} \frac{1}{N}\sum_{\ell=1}^{N} |w \cdot \phi(\boldsymbol{x}_\ell) - \mathrm{Tr}[\rho(\boldsymbol{x}_\ell)O(\boldsymbol{\alpha})]|^2 \tag{20}$$

Because we have set $B = \mathrm{poly}(n)$, we have that the second term must be at most $\hat{R}(h_{opt})$ and therefore we have

$$\hat{R}(h) \leq (B\epsilon_1 + \epsilon_2)^2 + \frac{\epsilon_3}{2} \tag{21}$$

We note that as $B \leq \mathcal{O}(\mathrm{poly}(n))$ we can bound the error $\epsilon_1' = B\epsilon_1$ to scale polynomially to zero by just reducing the sampling error ϵ_1 using polynomially many more copies of each $\rho(\boldsymbol{x}_l)$. Thus we can rewrite equation (13) as

$$R(h) \leq (\epsilon_1' + \epsilon_2)^2 + \frac{\epsilon_3}{2} + 2BM\sqrt{\frac{2\log(2m)}{N}} + M^2\sqrt{\frac{\log(\delta)^{-1}}{2N}} \tag{22}$$

Then, in order to bound the prediction error above by $\epsilon = (\epsilon_1' + \epsilon_2)^2 + \epsilon_3$ we need to choose an N such that

$$2BM\sqrt{\frac{2\log(2m)}{N}} + M^2\sqrt{\frac{\log(\delta)^{-1}}{2N}} \leq \frac{\epsilon_3}{2} \tag{23}$$

By substituting $M = B$ we obtain that:

$$2BM\sqrt{\frac{2\log(2m)}{N}} + M^2\sqrt{\frac{\log(\delta)^{-1}}{2N}} \leq \left(\frac{B^2}{\sqrt{2N}}(4\sqrt{\log(2\mathcal{O}(\mathrm{poly}(n)))} + \sqrt{\log(\delta^{-1})}\right) \tag{24}$$

so that it is upper bounded by $\epsilon_3/2$ choosing N as

$$N = 2\frac{B^4\sqrt{2\log(\mathcal{O}(\mathrm{poly}(n))/\delta)}}{(\epsilon_3)^2} \tag{25}$$

$$\sim \frac{\log(\mathrm{poly}(n)/\delta)\mathrm{poly}(n)}{(\epsilon_3)^2} \tag{26}$$

By setting $\epsilon_1' = 0.2\epsilon$, $\epsilon_2 = \epsilon$, and $\epsilon_3 = 0.4\epsilon$ we have $(\epsilon_1' + \epsilon_2)^2 + \epsilon_3 \leq \epsilon$ and recover the claim of the Theorem.

Finally we bound the efficiency of our algorithm. The training time is dominated by the creation of the feature map $\phi(\boldsymbol{x}_\ell)$ for each training point and by the LASSO regression over the corresponding

feature space. To create the vector $\phi(\boldsymbol{x})$ the quantum algorithm needs to prepare multiple copies of $\rho(\boldsymbol{x}_\ell)$ $\forall \boldsymbol{x}_\ell \in T_N^{\boldsymbol{\alpha}}$. As seen before, only a polynomial number of copies are sufficient to achieve a desired error ϵ_1', so that the whole process takes time $\mathcal{O}(\text{poly}(n)N)$. For the LASSO regression, it is known that to obtain a training error at most $\epsilon_3/2$ larger than the optimal function value, the LASSO algorithm on the feature space of $\phi(\boldsymbol{x})$ can be executed in time $\mathcal{O}\left(\frac{m_\phi \log m_\phi}{\epsilon_3^2}\right)$ [15], where in our case $m_\phi = m$. It is easy to show that even this time is bounded by $\mathcal{O}(\text{poly}(n)N)$. The prediction time corresponds to the time for the evaluation of the learned model $h(\boldsymbol{x}) = w^* \cdot \phi(\boldsymbol{x})$ which takes time $\mathcal{O}(m) \sim \mathcal{O}(\text{poly}(n))$. In conclusion, the overall time of the quantum algorithm is bounded by $\mathcal{O}(\text{poly}(n)N)$

□

5 Construction of the Kitaev's circuit-to-Hamiltonian for the ground state

To make our work more self-contained, we report the construction of the Kitaev's circuit-to-Hamiltonian which was used in the proof of Theorem 2. We follow the constructions outlined in [16, 17]. Let $x \in \mathcal{L}$ an input of a language $\mathcal{L} \in \mathsf{BQP}$ and $U(x) = U_T ... U_1$ be a quantum circuit of polynomial size T acting on $n = \text{poly}(|x|)$ qubits which decides x. We first add $2T$ layers of identities to $U(x)$. The Hamiltonian H that is constructed operates on a space of $n = N + \log(3T+1)$ qubits. The first N qubits represent the computation, and the last $\log(3T+1)$ qubits represent the possible values $0, \ldots, 3T$ for the clock. The Hamiltonian is constructed from three terms:

$$H(x) = H_{\text{init}} + H_{\text{clock}} + \sum_{t=1}^{3T} H_t(x), \tag{27}$$

with

$$H_{\text{init}} = \sum_{i=1}^{N} |0\rangle \langle 0|_i \,, \quad H_{\text{clock}} = \sum_{t=1}^{3T-1} |01\rangle \langle 01|_{t,t+1}^{\text{clock}} \,, \tag{28}$$

$$H_t(x) = \frac{1}{2}\Big(I \otimes |100\rangle \langle 100|_{t-1,t,t+1}^{\text{clock}} \tag{29}$$

$$+ I \otimes |110\rangle \langle 110|_{t-1,t,t+1}^{\text{clock}} \tag{30}$$

$$- U_t(x) \otimes |110\rangle \langle 100|_{t-1,t,t+1}^{\text{clock}} \tag{31}$$

$$- U_t(x)^\dagger \otimes |100\rangle \langle 110|_{t-1,t,t+1}^{\text{clock}} \Big) \tag{32}$$

where $|.\rangle \langle .|_i$ acts on the ith site of $\mathbb{C}^{2^N}$, $|.\rangle \langle .|_j^{\text{clock}}$ acts on the jth site of $\mathbb{C}^{2^{3T}}$ and U_t denotes the tth layer of gates in $U(x)$. Note that $H(x)$ is 5-local for all x. The ground state of $H(x)$ is given by $\rho(x) = |\psi(x)\rangle \langle \psi(x)|$, where

$$|\psi(x)\rangle = \frac{1}{\sqrt{3T}} \sum_{t=1}^{3T} (U_t \cdots U_1)(x) \, |0^N\rangle \, |1^t 0^{3T-t}\rangle \,, \tag{33}$$

Measuring the local observable $O = Z \otimes I \otimes |1\rangle \langle 1|_T^{\text{clock}}$ on the state $|\psi(x)\rangle$ will decide the input x, analogously to the case in the proof of Theorem 4.

6 Proof of Theorem 3 and Corollary 1 in the main text

We rewrite here Theorem 3 form the main text, formalizing the result and providing a detailed proof.

Theorem 3 (Theorem 3 in the main text)**.** *Assuming that* BQP $\not\subseteq$ P/poly*, the following holds. Let* $S = \{|\psi_\ell\rangle\}_\ell$ *and* $Q = \{Q_m\}_m$ *be a discrete set of quantum states and measurement operators, respectively, both of which are polynomially describable. Then for every efficient (non-adaptive) algorithm* $\mathcal{A}_W$ *which learns an arbitrary unitary* W *from query access to it on states from* S *and measured by operators in* Q *there exists a set of distributions* $\{\mathcal{D}_i\}_i$ *over* $\{0,1\}^n$*, a family of unitaries* $\{U(\boldsymbol{x})\}_{\boldsymbol{x}}$ *and a measurement* O *for which the concept class* $\mathcal{M}_{U,W,O}$ *exhibits a learning advantage under the learning condition of Def. 4.*

Proof. We can think at the algorithm $\mathcal{A}_W$ as an algorithm which receives in input pairs of $\{(|\psi_\ell\rangle, y_\ell^{m_\ell})_\ell\}$, where $|\psi_\ell\rangle \in S$ and $y_\ell^{m_\ell} \in \mathbb{R}$ is the measurement outcome of a randomly selected operator $Q_{m_l} \in Q$ on $|\psi_\ell\rangle$, and for any $\epsilon \geq 0$ outputs a matrix $\tilde{W}$ such that $||\tilde{W} - W|| \leq \epsilon$ in some norm distance. The idea to construct a $\mathcal{M}_{U,W,O}$ which exhibits a separation is the following, illustrated also in Figure 1. Consider the family of quantum states $\{|\psi_U(\boldsymbol{x})\rangle\}_{\boldsymbol{x}}$ constructed by parameterized unitaries $\{U(\boldsymbol{x})\}_{\boldsymbol{x}}$ in the following way, depending on the input $\boldsymbol{x} \in \{0,1\}^n$:

- The first $1+n_Q$ qubit of $|\psi_U(\boldsymbol{x})\rangle$ are initializated in the state $|x_1\rangle \otimes |x_2 x_3 ... x_{n_Q+1}\rangle$, the remaining $n_S = n - (1 + n_Q)$ qubit $\boldsymbol{x_S} = x_{n-n_S} x_{n-(n_S-1)} ... x_n$ are in the state $|0^{\otimes n_S}\rangle$.
- If the first bit x_1 is 0, then $U(\boldsymbol{x})$ prepares on the n_S qubits register the state $|\psi_{\boldsymbol{x_S}}\rangle$ from the set S described by the bitstring $\boldsymbol{x_S} \in \{0,1\}^{n_S}$. As we assumed that every state in S allows a polynomial classical description, we only need an input $\boldsymbol{x} \in \{0,1\}^n$ of polyinomial size. We thus have:
$$U(\boldsymbol{x}) \left(|0\rangle \otimes |\boldsymbol{x_Q}\rangle \otimes |0^{\otimes n_S}\rangle\right) = |0\rangle \otimes |\boldsymbol{x_Q}\rangle \otimes |\psi_{stab}(\boldsymbol{x_S})\rangle \tag{34}$$
- If the first bit x_1 is 1, then on the n_S qubit register $U(\boldsymbol{x})$ prepares the state $|\psi_{BQP}(\boldsymbol{x_S})\rangle$ such that $\langle\psi_{BQP}(\boldsymbol{x_S})| Z \otimes I \otimes ... \otimes I |\psi_{BQP}(\boldsymbol{x_S})\rangle$ outputs $+1/3$ if the n_S bitstring $\boldsymbol{x_S}$ belongs to an arbitrary (previously fixed) BQPcomplete language $\mathcal{L}$ defined over input $\boldsymbol{x_S} \in \{0,1\}^{n_S}$, while it outputs $-1/3$ otherwise. Following the same arguments used in the proof of Theorem 4, for any $\mathcal{L} \in$ BQP it always exists such $U(\boldsymbol{x})$. We thus have:
$$U(\boldsymbol{x}) \left(|1\rangle \otimes |\boldsymbol{x_Q}\rangle \otimes |0^{\otimes n_S}\rangle\right) = |1\rangle \otimes |\boldsymbol{x_Q}\rangle \otimes |\psi_{BQP}(\boldsymbol{x_S})\rangle \tag{35}$$

Regarding the unknown observable $O(\boldsymbol{\alpha})$, we will consider a measurement operator of the kind Eq.(6). Specifically we define a controlled operator V_A, controlled by the $1 + n_Q$ qubit register $|x_1 x_2 x_3 ... x_{n_Q+1}\rangle$, such that $V_A = \sum_{0\boldsymbol{x_Q}} |0\boldsymbol{x_Q}\rangle\langle 0\boldsymbol{x_Q}| \otimes V(\boldsymbol{x_Q})$ with $\boldsymbol{x_Q} = x_2 x_3 ... x_{n_Q+1}$ if $x_1 = 0$ and $V_A = \sum_{1\boldsymbol{x_Q}} |1\boldsymbol{x_Q}\rangle\langle 1\boldsymbol{x_Q}| \otimes I^{\otimes n_S}$ if $x_1 = 1$. The unitary matrices $V(\boldsymbol{x_Q})$ are defined such that they rotate the measurement O into the observable $Q_{\boldsymbol{x_Q}} \in Q$ described by the bitstring $\boldsymbol{x_Q} = x_2 x_3 ... x_{n_Q+1}$, i.e. $Q_{\boldsymbol{x_Q}} = V(\boldsymbol{x_Q}) O V^\dagger(\boldsymbol{x_Q})$ where O could be taken as the local observable $Z_1 = Z \otimes I^{\otimes n_S - 1}$ on the n_S qubit register. The final observable $O(\boldsymbol{\alpha})$ will then be $O(\boldsymbol{\alpha}) = (I^{\otimes(1+n_Q)} \otimes W(\boldsymbol{\alpha})\ V_A) O (V_A\ I^{\otimes(1+n_Q)} \otimes W(\boldsymbol{\alpha}))^\dagger$, with $W(\boldsymbol{\alpha})$ an arbitrary unitary parameterized by $\boldsymbol{\alpha}$. Furthermore, we define $W(\boldsymbol{\alpha} = \vec{0}) = I \otimes ... \otimes I$. We can now construct a set of distributions $\{\mathcal{D}_i\}_i$ such that the concept class $\mathcal{M}_{U,W,O}$ exhibits a learning separation. For Theorem 4, under the learning condition in Def. 4, the concept $f^0 = \mathrm{Tr}[\rho_U(\mathbf{x}) V_A O V_A^\dagger]$ cannot be efficiently learned by a classical algorithm when $U(\boldsymbol{x})$ is the circuit which decides the BQP-complete language $\mathcal{L}$ over the n_S-sized bitstrings $\boldsymbol{x_S}$. We now define the following set distributions $\{\mathcal{D}_i\}_i$ on the input bitsrings $\boldsymbol{x} \in \{0,1\}^n$:

- The first bit x_1 of $\boldsymbol{x}$ is extracted randomly with equal probability between 0 or 1.
- If $x_1 = 0$ then the other $n-1$ bit $x_2 x_3 ... x_n$ are extracted from the required distribution by $\mathcal{A}_W$ to learn the unitary $W(\boldsymbol{\alpha})$.
- If $x_1 = 1$ then the following n_Q bit $\boldsymbol{x_Q}$ are extracted uniformly at random while the n_S bit $\boldsymbol{x_S}$ are sampled with an arbitrary input distribution over $\boldsymbol{x_S} \in \{0,1\}^{n_S}$ which specifies the overall distribution $\mathcal{D}_i$ for each i.

Classical hardness The classical hardness of the learning task comes directly from the proof of Theorem 4. Consider the concept $f^0 \in \mathcal{M}_{U,W,M}$ defined by $W(\mathbf{0}) = I^{\otimes n_S}$. For the same reasoning of the proof of Theorem 4 there cannot exists a polynomial sized classical circuit which evaluates $f^0(\boldsymbol{x})$. Since for any of the input distributions $\mathcal{D}_i$, x_1 is equally sampled between 0 and 1, no classical algorithm can meet the learning condition of Eq. 4 on half of the input bitstrings, thus it can not learn the concept $f^0 \in \mathcal{M}_{U,W,O}$ in polynomial time for every ϵ. As the learning algorithm must suceed for every $\boldsymbol{\alpha}$, this suffices to prove the classical hardness of the learning task.

Quantum learnability Recall that the data the learning algorithm receives for a concept $f^{\boldsymbol{\alpha}} \in \mathcal{M}_{U,W,M}$ are $\mathcal{T}^{\boldsymbol{\alpha}} = \{\boldsymbol{x}_\ell, y_\ell\}_\ell$ with $\mathbb{E}[y_\ell] = \mathrm{Tr}[\rho_U(\boldsymbol{x}_\ell)O(\boldsymbol{\alpha})]$ and $\rho_U(\boldsymbol{x}_\ell) = |\psi_U(\boldsymbol{x})\rangle\langle\psi_U(\boldsymbol{x})|$. Now, in the case the first bit of $\boldsymbol{x}_\ell$ is 0, $\mathcal{T}^{\boldsymbol{\alpha}}$ are exaclty the pairs $\{(|\psi_\ell\rangle, y_\ell^m)\}_\ell$ required by the algorithm $\mathcal{A}_W$ to learn $W(\boldsymbol{\alpha})$ and thus $O(\boldsymbol{\alpha})$. As $x_1 = 0$ for half of the training samples in $\mathcal{T}^{\boldsymbol{\alpha}}$, the quantum algorithm is able to learn $O(\boldsymbol{\alpha})$ and evaluate it on any input state.

□

We now provide here the the rigorous proof of Corollary 1. The proof follows the same steps of the one for Theorem 3 above while concretizing the result for shallows W.

First, let us repeat the result in [18] for learning shallow unitaries. Be stab1 the family of single qubit stabilizer states stab1 $= \{|0\rangle, |1\rangle, |+\rangle, |-\rangle, |y+\rangle, |y-\rangle\}$, then:

Lemma 3 (Lemma 10 in [18], Learning a few-body observable with an unknown support)**.** *Given an error ϵ, failure probability δ, an unknown n-qubit observable O with $\|O\|_\infty \leq 1$ that acts on an unknown set of k qubits, and a dataset $\mathcal{T}_O(N) = \{|\psi_\ell\rangle = \bigotimes_{i=1}^n |\psi_{\ell,i}\rangle, v_\ell\}_{\ell=1}^N$, where $|\psi_{\ell,i}\rangle$ is sampled uniformly from* stab1 *and v_ℓ is a random variable with $E[v_\ell] = \langle\psi_\ell|O|\psi_\ell\rangle$, $|v_\ell| = \mathcal{O}(1)$. Given a dataset size of*

$$N = \frac{2^{\mathcal{O}(k)}\log(n/\delta)}{\epsilon^2}, \tag{36}$$

with probability at least $1-\delta$, we can learn an observable O' such that $\|O'-O\|_\infty \leq \epsilon$ and $\mathrm{supp}(O') \subseteq \mathrm{supp}(O)$*. The computational complexity is $\mathcal{O}(n^k \log(n/\delta)/\epsilon^2)$.*

The proof of Corollary 1 then goes by explicitly constructing a family of parametrized circuit $\{U(\boldsymbol{x})\}_{\boldsymbol{x}}$ and a set of input distributions $\{\mathcal{D}_i\}_i$ such that the concept class $\mathcal{M}_{U,W,M}$ is quantum learnable using Lemma 3 while still being classically hard. Let us rewrite here Corollary 1

Corollary 1 (Learning advantage for shallow unitaries)**.** *corollaryshallowadv There exists a family of parametrized unitaries $\{U(\boldsymbol{x})\}_x$ and parametrized shallow circuits $\{W(\boldsymbol{\alpha})\}_{\boldsymbol{\alpha}}$, a measurement O and a set of distributions $\{\mathcal{D}_i\}_i$ over $\boldsymbol{x} \in \{0,1\}^n$ such that the concept class $\mathcal{M}_{U,W,O}$ is not classically learnable with respect to the set of input distributions $\{\mathcal{D}_i\}_i$, unless* $\mathsf{BQP} \subseteq \mathsf{P/poly}$*. However, there exists a quantum algorithm which learns $\mathcal{M}_{U,W,O}$ on the input distributions $\{\mathcal{D}_i\}_i$.*

Proof. Let first define the set of unitaries $\{U(\boldsymbol{x})\}_{\boldsymbol{x}}$, $\{W(\boldsymbol{\alpha})\}_{\boldsymbol{\alpha}}$, measurement operator O and input distribution $\mathcal{D}$ on which the separation result holds. We provided a graphical representation of it in Supplementary figure 1. Be $n_S = \lfloor \frac{n}{3} \rfloor$, we define the set $\{U(\boldsymbol{x})\}_{\boldsymbol{x}}$ as the set of unitaries which act on a $n_S + 1$ qubit system in the following way, depending on the first bit x_1 of the input $\boldsymbol{x}$.

If $x_1 = 0$ then each $U(\boldsymbol{x})$ is defined such that

$$U(\boldsymbol{x})\left(|x_1\rangle \otimes |0^{n_S}\rangle\right) = |x_1\rangle \otimes |\psi_{stab}(\boldsymbol{x})\rangle \tag{37}$$

where $|\psi_{stab}(\boldsymbol{x})\rangle = \bigotimes_{i=1}^{n_S} |\psi^i_{stab1}(\boldsymbol{x})\rangle$ is a n_S qubit tensor product of single qubit stabilizer states $|\psi^i_{stab1}(\boldsymbol{x})\rangle \in \{|0\rangle, |1\rangle, |+\rangle, |-\rangle, |y+\rangle, |y-\rangle\}$. Since $n_S = \lfloor \frac{n}{3} \rfloor$, the output state $|\psi_{stab}(\boldsymbol{x})\rangle$ is completely described by the remaining $n-1$ input bits $x_2 x_3 \ldots x_n$.

Let consider now the case when $x_1 = 1$. Be $\mathcal{L}$ a BQP-complete language defined over the input $\tilde{\boldsymbol{x}} \in \{0,1\}^{n_S}$. Then we define the set of $\{U(\boldsymbol{x})\}_{\boldsymbol{x}}$ in the following way:

$$U(\boldsymbol{x})\left(|x_1\rangle \otimes |0^{n_S}\rangle\right) = |x_1\rangle \otimes |\psi_{BQP}(\boldsymbol{x})\rangle \tag{38}$$

where $|\psi_{BQP}(\boldsymbol{x})\rangle$ is such that $\langle\psi_{BQP}(\boldsymbol{x})|\, Z \otimes I \otimes ... \otimes I\, |\psi_{BQP}(\boldsymbol{x})\rangle = 1/3$ if $\tilde{\boldsymbol{x}} \in \mathcal{L}$ and $-1/3$ if $\tilde{\boldsymbol{x}} \notin \mathcal{L}$. As we described in the proof of Theorem 4, such a circuit $U_{BQP}(\boldsymbol{x})$ always exists for any BQP language.

As a set of unitaries $\{W(\boldsymbol{\alpha})\}_{\boldsymbol{\alpha}}$ we consider any set of parametrized unitaries acting on the n_S qubit register such that for each $\boldsymbol{\alpha}$ parameter $W(\boldsymbol{\alpha})$ is shallow and such that $W(\mathbf{0}) = I^{\otimes n_S}$. The n_S qubit register is then measured by the observable $M = Z \otimes I \otimes ... \otimes I$. Finally we define the set of distributions $\{\mathcal{D}_i\}_i$ on the input bitstrings $\boldsymbol{x} \in \{0,1\}^n$ to be the following:

- The first bit $x_1 \in \{0,1\}$ of $\boldsymbol{x}$ is extracted randomly with equal probability.
- If $x_1 = 0$ then the other $n-1$ bit $x_2x_3...x_n$ are extracted following the uniform distribution over $\{0,1\}^{n-1}$.
- If $x_1 = 1$ then the following n_S bits $x_2x_3...x_{n_S+1}$ are extracted from an arbitrary distribution which characterizes $\mathcal{D}_i$ for each index i. The other $n-(n_S+1)$ input bits are sampled uniformly at random.

We now show that the concept class $\mathcal{M}_{U,W,M}$ defined in Eq. 6 with $\{U(\boldsymbol{x})\}_{\boldsymbol{x}}$, $\{W(\boldsymbol{\alpha})\}_{\boldsymbol{\alpha}}$ and M considered above exhibits a learning separation with respect to the learning condition in Def. 4.

Classical hardness The argument is exactly the same as the one presented before in the proof of Theorem 3.

Quantum learnability The quantum learnability is guaranteed by Lemma 3 in [18]. Recall that the training data the learning algorithm receives for a concept $f^{\boldsymbol{\alpha}} \in \mathcal{M}_{U,W,M}$ are $\mathcal{T}^{\boldsymbol{\alpha}} = \{\boldsymbol{x}_\ell, y_\ell\}_\ell$ with $\mathbb{E}[y_\ell] = \mathrm{Tr}[\rho_U(\boldsymbol{x}_\ell)O(\boldsymbol{\alpha})]$. Now, in the case the first bit of $\boldsymbol{x}_\ell$ is 0, $\mathcal{T}^{\boldsymbol{\alpha}}$ is exactly the training set $\mathcal{T}_{\boldsymbol{\alpha}}$ required by the algorithm in Lemma 3 to learn $O(\boldsymbol{\alpha})$. Since for every $\boldsymbol{\alpha}$ the unitary $W(\boldsymbol{\alpha})$ is of shallow depth, the locality of $O(\boldsymbol{\alpha})$ scales logarithmic with the number of qubit n_S. Then Lemma 3 guarantees that the learning algorithm runs in polynomial time requiring a polynomial-sized dataset, a condition met as half of the training samples in $\mathcal{T}^{\boldsymbol{\alpha}}$ will suffice.

□

References

[1] Casper Gyurik and Vedran Dunjko. Exponential separations between classical and quantum learners. *arXiv preprint arXiv:2306.16028*, 2023.

[2] Hsin-Yuan Huang, Richard Kueng, Giacomo Torlai, Victor V Albert, and John Preskill. Provably efficient machine learning for quantum many-body problems. *Science*, 377(6613):eabk3333, 2022.

[3] Laura Lewis, Hsin-Yuan Huang, Viet T Tran, Sebastian Lehner, Richard Kueng, and John Preskill. Improved machine learning algorithm for predicting ground state properties. *nature communications*, 15(1):895, 2024.

[4] Emilio Onorati, Cambyse Rouzé, Daniel Stilck França, and James D Watson. Efficient learning of ground & thermal states within phases of matter. *arXiv preprint arXiv:2301.12946*, 2023.

[5] Emilio Onorati, Cambyse Rouzé, Daniel Stilck França, and James D Watson. Provably efficient learning of phases of matter via dissipative evolutions. *arXiv preprint arXiv:2311.07506*, 2023.

[6] Hayata Yamasaki, Natsuto Isogai, and Mio Murao. Advantage of quantum machine learning from general computational advantages. *arXiv preprint arXiv:2312.03057*, 2023.

[7] Hsin-Yuan Huang, Richard Kueng, and John Preskill. Information-theoretic bounds on quantum advantage in machine learning. *Physical Review Letters*, 126(19):190505, 2021.

[8] Abhishek Jain. Modern cryptography: Lecture notes. *Johns Hopkins University*, 2017. URL https://www.cs.jhu.edu/~abhishek/classes/CS600-642-442-Fall2019/notes.pdf?utm_source=chatgpt.com.

[9] Scott Aaronson. Aaronson's response to a question on slackexchange. URL https://cstheory.stackexchange.com/questions/15066/consequences-of-bqp-subseteq-p-poly.

[10] Andrew MacGregor Childs. *Quantum information processing in continuous time*. PhD thesis, Massachusetts Institute of Technology, 2004.

[11] Richard Feynman. Quantum mechanical computers. *Optics news*, 11(2):11–20, 1985.

[12] Daniel Nagaj. Fast universal quantum computation with railroad-switch local hamiltonians. *Journal of Mathematical Physics*, 51(6), 2010.

[13] Robert E Schapire. The strength of weak learnability. *Machine learning*, 5:197–227, 1990.

[14] Mehryar Mohri, Afshin Rostamizadeh, and Ameet Talwalkar. *Foundations of machine learning*. MIT press, 2018.

[15] Elad Hazan and Tomer Koren. Linear regression with limited observation. *arXiv preprint arXiv:1206.4678*, 2012.

[16] Alexei Yu Kitaev, Alexander Shen, and Mikhail N Vyalyi. *Classical and quantum computation*. Number 47. American Mathematical Soc., 2002.

[17] Julia Kempe and Oded Regev. 3-local hamiltonian is qma-complete. *arXiv preprint quant-ph/0302079*, 2003.

[18] Hsin-Yuan Huang, Yunchao Liu, Michael Broughton, Isaac Kim, Anurag Anshu, Zeph Landau, and Jarrod R McClean. Learning shallow quantum circuits. *arXiv preprint arXiv:2401.10095*, 2024.

Supplementary Figures

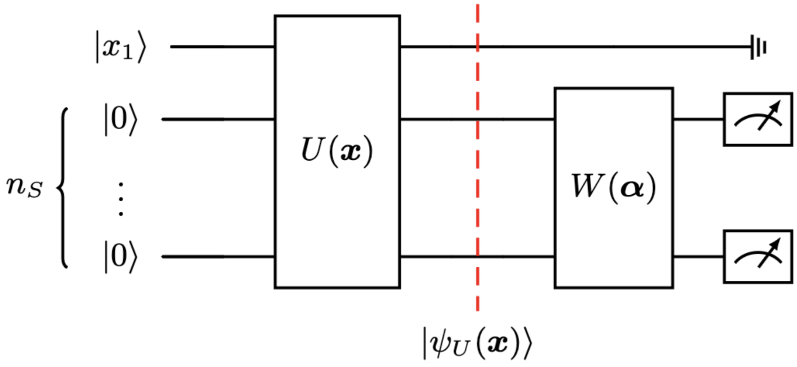

Supplementary figure 1: **A quantum model showing a learning separation for shallow-unitary–parameterized observables.** The unitary $U(\boldsymbol{x})$ prepares the state $|\psi_U(\boldsymbol{x})\rangle = |x_1\rangle \otimes |\psi_U^{n_S}(\boldsymbol{x_S})\rangle$ where the form of $|\psi_U^{n_S}(\boldsymbol{x_S})\rangle$ depends on x_1, the first bit of each input $\boldsymbol{x} \in \{0,1\}^n$. If $x_1 = 0$, then $|\psi_U^{n_S}(\boldsymbol{x_S})\rangle = |\psi_{stab}(\boldsymbol{x_S})\rangle = \bigotimes_{i=1}^{n_S} |\psi_{stab1}^{i}(\boldsymbol{x_S})\rangle$ is the $n_S = \lfloor \frac{n}{3} \rfloor$ qubit tensor product of single-qubit stabilizers described by the classical bitstring $x_2x_3...x_n$. If $x_1 = 1$, then $|\psi_U^{n_S}(\boldsymbol{x_S})\rangle$ is the quantum state which decides the n_S input bits $\boldsymbol{x_S} = x_2x_3...x_{n_S+1}$, considered as input of a BQP-complete language $\mathcal{L}$ over the bitstrings $\boldsymbol{x_S} \in \{0,1\}^{n_S}$. $W(\boldsymbol{\alpha})$ is a parametrized shallow unitary, to prove classical hardness it is sufficient to consider $W(\boldsymbol{\alpha} = 0) = I^{\otimes n_S}$ and the measurement operator to be $O = Z \otimes I \otimes ... \otimes I$ on the n_S qubits register.
